# Supplementary material for: Recognition of Serious Infections in the Elderly Visiting the Emergency Department: The Development of a Diagnostic Prediction Model (ROSIE)
Source: Geriatrics (Basel). 2025 Apr 25;10(3):60. doi: 10.3390/geriatrics10030060 (PMC12101360; doi:10.3390/geriatrics10030060)
Supplement: Supplementary file 1 [file geriatrics-10-00060-s001.zip › Appendix B Sample size and interim analysis_revised.pdf]

Appendix B: Sample size, interim analysis and emergency department characteristics

The ROSIE study was initially designed to be conducted in primary care, with additional recruitment in the ED as a contingency plan. Slow recruitment in primary care during the COVID-19 pandemic forced us to focus only on the ED setting, where the recruitment rate was much higher. The study arm in primary care was abandoned after only 66 participants were included over two years. These participants were not included in the analyses presented.

Original and final sample size

The original calculation of the sample size per protocol was as follows: "Setting the number of candidate predictors at 12 (10 predictors + 2 Restricted Cubic Splines), the events fraction at 4% (= expected incidence of a serious infection in the study population) and the criterion value of the square root of the mean squared prediction error (rMSPE) at 5%, the minimally required sample size is estimated to be 900 older adults. The rMSPE is based on the distance between the estimated probabilities and the true probabilities by the square root of the average squared distance. Lower values for rMSPE indicate better performance." This was calculated with the method from van Smeden et al (https://mvansmeden.shinyapps.io/BeyondEPV/).[1]

The protocol included a pre-planned interim analysis after the inclusion of the first 300 participants, to eliminate predictors based on futility. We operationalized futility as a univariable AUROC <0.60. For practical reasons (availability of the expert panel at that time), the interim analysis was eventually conducted after inclusion of 342 participants. The incidence of serious infections at the ED was 45% at the interim analysis, much higher than what we had originally estimated in primary care. The futility analysis reduced the number of predictors from 12 to 4. Re-estimating sample size using the same approach as above resulted in a final required sample size of 425. Consequently, as the interim analysis was conducted quite late (the sample size at the interim analysis is close to the final sample size), we decided to include this interim analysis in the internal validation process using bootstrapping.

Results of the interim analysis

Based on a combination of clinical reasoning and the results of the univariable analyses, the expert panel decided that following candidate predictors should be retained as predictors for the final model (Table S1): systolic blood pressure, peripheral oxygen saturation, C-reactive protein and procalcitonin. These four predictors were the only candidate predictors with an AUROC greater than 0.60. Procalcitonin was seen by the expert panel as a biomarker of second choice compared to C-reactive protein, due to its higher cost and its lower availability in EDs in Belgium. It would therefore have to demonstrate very substantial added value before it would be included in the final prediction model.

The expert panel also concluded that elevated body temperature (fever) and respiratory rate (tachypnoea) may be useful as red flags because of their high specificity, but are less suitable as predictors in a prediction model due to their low sensitivities at commonly used cutoff values (too many False Negatives). These predictors were therefore considered less relevant at the ED setting.

Table S1: Univariate AUROC analysis of candidate predictors *at interim* (n=342 unless specified otherwise)

Candidate predictor	AUROC[1]	95%Confidence Interval
Age (years)	0.51	0.45 to 0.57
Body temperature (°C)	0.57	0.50 to 0.63
Heart rate (beats/minute)	0.54	0.48 to 0.60
Respiratory rate (breaths/minute)	0.58	0.52 to 0.64
Systolic blood pressure (mmHg)	0.62	0.56 to 0.68
Peripheral oxygen saturation (SpO2, %)[2]	0.65	0.60 to 0.71
Level of confusion (CAM-S score)[3]	0.52	0.48 to 0.55
C-reactive protein (mg/L)[4], n=274	0.72	0.66 to 0.78
Procalcitonin (ng/mL)[4], n=276	0.70	0.64 to 0.76
Abnormal White Blood Cell Count[5], n=271	0.56	0.50 to 0.62

[1]AUROC = Area Under the Receiver Operating Characteristic; [2]Log(101-Oxygen saturation); [3]CAM-S = Confusion Assessment Method - Short form; [4]Log_2 transformed; [5]Abnormal White Blood Cell Count: <4000 or >12000 cells/mm³

The participating hospital emergency departments

1. **University Hospitals Leuven (UZ Leuven) - Gasthuisberg Campus**

University Hospitals Leuven (UZ Leuven) is one of the largest and most comprehensive academic medical centers in Belgium. It is closely affiliated with the KU Leuven university. The Gasthuisberg campus is the main and largest site of UZ Leuven, housing a wide range of specialized services and serving as a tertiary referral center for complex medical conditions. Seventy-eight percent of all inclusions were conducted at this ED (n=320). Its emergency department had 72,097 visits in the year 2022. The hospital has 1949 beds and is the largest university hospital in Belgium.

Type of Hospital and Structure:

- **Type:** UZ Leuven, with Gasthuisberg as its primary campus, is a university-affiliated tertiary care hospital. This means it provides highly specialized medical care, conducts cutting-edge research, and is involved in the education of future healthcare professionals.
- **Structure:**
  - UZ Leuven is organized into various departments and divisions, each specializing in a specific medical field (e.g., cardiology, oncology, infectious diseases, surgery).

- The Gasthuisberg campus is a large, multi-building complex. It includes:
    - Emergency Department: Provides 24/7 acute care services.
    - Inpatient wards: For patients requiring hospitalization.
    - Outpatient clinics: For consultations, treatments, and follow-up care.
    - Operating rooms: For surgical procedures.
    - Intensive care units (ICUs): For critically ill patients.
    - Diagnostic and support services: Including laboratories, imaging departments, pharmacy, and rehabilitation services.

- The Emergency Department at Gasthuisberg is a high-volume, complex environment with specialized areas for trauma, medical emergencies, and observation. It operates with a multidisciplinary team, including emergency physicians, nurses, and other specialists.
- As a university hospital, there is a strong emphasis on a structured approach to care, often involving protocols and guidelines based on the latest evidence.

Infection Control Policies:

UZ Leuven, as a leading academic medical center, places a high priority on infection prevention and control. Key aspects of their infection control policies and practices likely include:

- **Comprehensive Infection Control Program:** A dedicated infection control team oversees and implements infection prevention strategies across the hospital. This team typically includes infection control physicians, nurses, and microbiologists.

  Standard Precautions: All healthcare workers adhere to standard precautions, including hand hygiene protocols, the use of personal protective equipment (PPE), safe injection practices, respiratory hygiene and cough etiquette, and safe handling of sharps and waste.

  Transmission-Based Precautions: In addition to standard precautions, transmission-based precautions are used for patients with known or suspected infections that can spread through contact, droplet, or airborne routes. These include contact precautions, droplet precautions and airborne precautions.

- **Surveillance and Monitoring:** UZ Leuven likely has robust systems for surveillance and monitoring of healthcare-associated infections (HAIs). This involves tracking infection rates, identifying outbreaks and analyzing trends to improve prevention strategies.
- **Antibiotic Stewardship Program:** To combat antimicrobial resistance, UZ Leuven likely has an antibiotic stewardship program that promotes the appropriate use of antibiotics.
- **Specific Infection Prevention Measures:**
    - Protocols for the prevention of specific HAIs, such as:
        - Catheter-associated urinary tract infections (CAUTI).
        - Central line-associated bloodstream infections (CLABSI).

        - Surgical site infections (SSI).
        - Ventilator-associated pneumonia (VAP).
    - Environmental cleaning and disinfection protocols.
    - Isolation procedures for patients with infectious diseases.
- **Education and Training:** Ongoing education and training for all healthcare workers on infection prevention and control practices.

2. Heilig Hart hospital Leuven

Heilig Hart Hospital Leuven is a general, 287-bed hospital providing a range of healthcare services to the Leuven region. While it may not have the same level of specialization or research focus as a university hospital like UZ Leuven, it plays a crucial role in delivering essential medical care to the local community. Eight percent of all inclusions were conducted at this ED (32/245). Its emergency department had 21,295 visits in the year 2022.

Type of Hospital and Structure:

- **Type:** Heilig Hart Hospital is a general hospital or regional hospital. This means it offers a broad spectrum of medical services.
- **Infection Control Policies:**
  All hospitals in Belgium are required to adhere to national and regional guidelines and regulations regarding infection prevention and control. Therefore, Heilig Hart Hospital has robust infection control policies and practices in place (Infection control team, standard precautions, transmission-based precautions, Healthcare-Associated Infection (HAI) surveillance, antibiotic stewardship, specific infection prevention measures, and staff education and training).

3. AZ Voorkempen, Malle, Belgium

AZ Voorkempen, located in Malle, is a regional, 250-bed hospital providing a range of healthcare services to the population in the Voorkempen region of Belgium. Seventeen percent of all inclusions were conducted at this ED (73/425). Its emergency department had 25,704 visits in the year 2022.

Type of Hospital and Structure:

- **Type:** AZ Voorkempen Hospital is a general hospital or regional hospital. This means it offers a broad spectrum of medical services.
- **Infection Control Policies:**
  All hospitals in Belgium are required to adhere to national and regional guidelines and regulations regarding infection prevention and control. Therefore, AZ Voorkempen Hospital has robust infection control policies and practices in place (Infection control team, standard precautions, transmission-based precautions, Healthcare-Associated

Infection (HAI) surveillance, antibiotic stewardship, specific infection prevention measures, and staff education and training).

References

1. van Smeden M, Moons KG, de Groot JA, et al. Sample size for binary logistic prediction models: Beyond events per variable criteria. *Stat Methods Med Res.* 2018:962280218784726.
2. https://jaarverslag.hhleuven.be/nl/#yearnumbers
3. https://www.azvoorkempen.be/over-ons/de-cijfers/activiteit
4. https://www.uzleuven.be/en/2022-key-figures
