# Supplementary material for: Recognition of Serious Infections in the Elderly Visiting the Emergency Department: The Development of a Diagnostic Prediction Model (ROSIE)
Source: Geriatrics (Basel). 2025 Apr 25;10(3):60. doi: 10.3390/geriatrics10030060 (PMC12101360; doi:10.3390/geriatrics10030060)
Supplement: Supplementary file 1 [file geriatrics-10-00060-s001.zip › Appendix C Information collected.pdf]

Appendix C: Information collected by the ROSIE study nurse

Variable name	Description	Unit of measurement
Candidate predictors: vital signs (measured at the time of inclusion)		
Date of birth	Age (calculated)	Years
Body temperature	Body temperature	°C
Heart rate	Heart rate	Beats per minute
Respiratory rate	Breathing frequency	Breaths per minute
Systolic blood pressure	Systolic blood pressure	mmHg
Oxygen saturation	Peripheral oxygen saturation	%
Level of confusion	Score on Confusion Assessment Method short form (CAM-S)	Score from 1 to 7
Candidate predictors: Blood tests (sample taken at the time of inclusion)		
CRP	C-reactive Protein	mg/L
PCT	Procalcitonin	ng/ml
WBC	White Blood cell Count	cells per µL
Other variables measured at the time of inclusion		
Participant Id, Site (ED), Participant Creation Date, Date of birth, Nursing home residency, Gender, Duration illness, Height, Method height measurement, Weight, Method weight measurement, BMI, Weight loss, Method of temperature measurement, Maximum body temperature, Subjective feeling fever, Diastolic blood pressure, Additional oxygen given, malaise, diarrhea, cough, headache, abdominal pain, falls, sputa, percussion pain kidney, pollakiuria, dysuria, dyspnea, lung crepitations, meningeal irritation, petechiae, abdominal irritation, other signs of infection, estimated severity, working hypothesis, antibiotics at inclusion, specification antibiotics, antipyretic medication, referral by GP, additional investigations, level of consciousness (Glasgow Coma), level of functional independence prior to inclusion (KATZ)		
Variables measured during 30-day follow-up		
level of functional independence 30 days after inclusion (KATZ by telephone), ED reconsultations, hospitalization, change in working hypothesis, final diagnosis, additional medication, additional investigations, complications, surgery, chronic medication, comorbidities, smoking status, vaccination status influenza, vaccination status pneumococci, vaccination status Sars-Cov-2		
