# Supplementary material for: Recognition of Serious Infections in the Elderly Visiting the Emergency Department: The Development of a Diagnostic Prediction Model (ROSIE)
Source: Geriatrics (Basel). 2025 Apr 25;10(3):60. doi: 10.3390/geriatrics10030060 (PMC12101360; doi:10.3390/geriatrics10030060)
Supplement: Supplementary file 1 [file geriatrics-10-00060-s001.zip › Appendix D Reference standards.pdf]

**Appendix D: Infections always regarded as serious and their reference standards**

- Pneumonia:[1-3]
  Infiltrate on chest radiograph or computed tomography (CT) within 7 days after inclusion
- Infectious acute exacerbation of COPD:[4]
  An acute worsening of symptoms such as dyspnoea, cough and/or sputum production in a patient with a spirometry confirmed COPD (post bronchodilator $FEV_1/FVC < 0.70$), resulting in hospital admission and anti-infectious treatment

- Influenza infection requiring hospital admission:[5]

  Confirmation of the presence of influenza virus (type A or B) in an upper respiratory tract specimen by the use of reverse transcription polymerase chain reaction (RT-PCR) in a hospitalised patient

- Severe Acute Respiratory Syndrome Corona virus (SARS-CoV-2) infection requiring hospital admission:

  Confirmation of the presence of SARS-CoV-2 infection in a nasopharyngeal specimen by the use of reverse-transcriptase polymerase-chain-reaction (RT-PCR) assay in a patient who was hospitalised because of a suspicion of SARS-CoV-2 infection.
- Sepsis:[6]
  Suspected or blood culture proven infection and an acute increase of ≥2 SOFA points (organ dysfunction), following the Sepsis-3 guidelines
- Complicated UTI:[7]
  Uropathogen growth of at least $10^5$ colony forming units/ml in urine culture, with clinical symptoms and signs attributable to a complicated UTI, or abnormal imaging results such as CT, DMSA scans or ultrasound
- Infectious encephalitis/meningitis:[8]
  Pleocytosis and pathogen identification from cerebrospinal fluid (viral or bacterial) via culture or PCR
- Osteomyelitis:[7]
  - Single pathogen from bone aspirate
    OR
  - radiographic imaging compatible with osteomyelitis combined with a positive blood culture
- Prosthetic joint infections:[9]

Radiolucency surrounding prosthesis on radiography

- Septic (infectious) arthritis:[10,11]
  Single pathogen from synovial fluid aspirate
- Infective endocarditis (IE):[12,13]
  A diagnosis of infective endocarditis is definite if the following pathological OR clinical criteria are met (modified Duke criteria):

  - One of these pathological criteria:

    - Histology or culture of a cardiac vegetation, an embolized vegetation, or intracardiac abscess from the heart finds microorganisms
    - Active endocarditis

  - One of these combinations of clinical criteria:
    - 2 major clinical criteria
    - 1 major and 3 minor criteria
    - 5 minor criteria
      (Major clinical criteria: Positive blood culture with typical IE microorganism, evidence of endocardial involvement with positive echocardiogram)
      (Minor clinical criteria: e.g. a predisposing factor, fever >38°C, immunological phenomena, microbiological phenomena…)

- Infectious cholecystitis:[14]
  - combination of clinical features such as right epigastric pain, a palpable gallbladder, and Murphy's sign, and positive findings on imaging: abdominal ultrasound or abdominal CT with intravenous contrast
    OR
  - pathological confirmation after surgery
- Infectious spondylitis or spondylodiscitis:[15]
  - Magnetic resonance imaging (MRI) of the spine.
  - If an MRI cannot be obtained: CT scan or a positron emission tomography (PET) scan.
- Appendicitis:[16]
  - Positive findings on CT or MRI. Ultrasound imaging serves as a second choice.
    OR
  - pathological confirmation after surgery
- Complicated diverticulitis:[17-22]

Clinical assessment including signs of peritonitis, ileus, rectal bleeding, local muscular resistance, hypotension with or without laboratory tests such as CRP>100 mg/L, followed by:

- CT-scan of abdomen and pelvis
  OR
- ultrasound imaging if CT not available
  OR
- pathological confirmation after surgery

- Cellulitis: acute, suppurated inflammation of the subcutaneous tissues

**References**


1. NICE. Pneumonia in adults: diagnosis and management (CG191). Clinical guideline. National Institute for Health and Care Excellence. https://www.nice.org.uk/guidance/cg191. Published 2014. Accessed.
2. Prendki V, Scheffler M, Huttner B, et al. Low-dose computed tomography for the diagnosis of pneumonia in elderly patients: a prospective, interventional cohort study. *The European respiratory journal.* 2018;51(5).
3. Haga T, Fukuoka M, Morita M, Cho K, Tatsumi K. Computed Tomography for the Diagnosis and Evaluation of the Severity of Community-acquired Pneumonia in the Elderly. *Internal medicine (Tokyo, Japan).* 2016;55(5):437-441.
4. GOLD. Global strategy for the diagnosis, management and prevention of chronic obstructive pulmonary disease: 2018 report. Global Initiative for Chronic Obstructive Pulmonary Disease, Inc. (GOLD). https://goldcopd.org/wp-content/uploads/2017/11/GOLD-2018-v6.0-FINAL-revised-20-Nov_WMS.pdf. Published 2018. Accessed 6 November 2018.
5. WHO. Manual for the laboratory diagnosis and virological surveillance of influenza. World Health Organization. Published 2011. Accessed.
6. Singer M, Deutschman CS, Seymour CW, et al. The Third International Consensus Definitions for Sepsis and Septic Shock (Sepsis-3). *JAMA.* 2016;315(8):801-810.
7. Yoshikawa TT, Norman DC. *Infectious Diseases in Geriatric Medicine, An Issue of Clinics in Geriatric Medicine.* Vol 32-3. 1st ed: Elsevier; 2016.
8. van de Beek D, Cabellos C, Dzupova O, et al. ESCMID guideline: diagnosis and treatment of acute bacterial meningitis. *Clin Microbiol Infect.* 2016;22 Suppl 3:S37-62.
9. Osmon DR, Berbari EF, Berendt AR, et al. Diagnosis and management of prosthetic joint infection: clinical practice guidelines by the Infectious Diseases Society of America. *Clin Infect Dis.* 2013;56(1):e1-e25.
10. Goldenberg DL. Septic arthritis. *Lancet.* 1998;351(9097):197-202.
11. Mathews CJ, Coakley G. Septic arthritis: current diagnostic and therapeutic algorithm. *Curr Opin Rheumatol.* 2008;20(4):457-462.
12. Habib G, Lancellotti P, Iung B. 2015 ESC Guidelines on the management of infective endocarditis: a big step forward for an old disease. *Heart.* 2016;102(13):992-994.

13. Li JS, Sexton DJ, Mick N, et al. Proposed modifications to the Duke criteria for the diagnosis of infective endocarditis. *Clin Infect Dis.* 2000;30(4):633-638.
14. Miura F, Takada T, Kawarada Y, et al. Flowcharts for the diagnosis and treatment of acute cholangitis and cholecystitis: Tokyo Guidelines. *Journal of hepato-biliary-pancreatic surgery.* 2007;14(1):27-34.
15. Berbari EF, Kanj SS, Kowalski TJ, et al. 2015 Infectious Diseases Society of America (IDSA) Clinical Practice Guidelines for the Diagnosis and Treatment of Native Vertebral Osteomyelitis in Adults. *Clin Infect Dis.* 2015;61(6):e26-46.
16. Gorter RR, Eker HH, Gorter-Stam MA, et al. Diagnosis and management of acute appendicitis. EAES consensus development conference 2015. *Surgical endoscopy.* 2016;30(11):4668-4690.
17. Ambrosetti P, Becker C, Terrier F. Colonic diverticulitis: impact of imaging on surgical management -- a prospective study of 542 patients. *Eur Radiol.* 2002;12(5):1145-1149.
18. Ambrosetti P, Grossholz M, Becker C, Terrier F, Morel P. Computed tomography in acute left colonic diverticulitis. *Br J Surg.* 1997;84(4):532-534.
19. Ambrosetti P, Jenny A, Becker C, Terrier TF, Morel P. Acute left colonic diverticulitis--compared performance of computed tomography and water-soluble contrast enema: prospective evaluation of 420 patients. *Dis Colon Rectum.* 2000;43(10):1363-1367.
20. O'Leary DP, Lynch N, Clancy C, Winter DC, Myers E. International, Expert-Based, Consensus Statement Regarding the Management of Acute Diverticulitis. *JAMA Surg.* 2015;150(9):899-904.
21. Vennix S, Morton DG, Hahnloser D, Lange JF, Bemelman WA, Research Committee of the European Society of C. Systematic review of evidence and consensus on diverticulitis: an analysis of national and international guidelines. *Colorectal Dis.* 2014;16(11):866-878.
22. Werner A, Diehl SJ, Farag-Soliman M, Duber C. Multi-slice spiral CT in routine diagnosis of suspected acute left-sided colonic diverticulitis: a prospective study of 120 patients. *Eur Radiol.* 2003;13(12):2596-2603.
