# Supplementary material for: Recognition of Serious Infections in the Elderly Visiting the Emergency Department: The Development of a Diagnostic Prediction Model (ROSIE)
Source: Geriatrics (Basel). 2025 Apr 25;10(3):60. doi: 10.3390/geriatrics10030060 (PMC12101360; doi:10.3390/geriatrics10030060)
Supplement: Supplementary file 1 [file geriatrics-10-00060-s001.zip › Appendix E Imputation of missing values.pdf]

Appendix E: Imputation of missing values.

First, we imputed abnormal blood cell count (7 missing values) using a logistic regression model with the following covariates: age, body temperature, the log2 transformed heart rate, the log2 transformed respiratory rate, the CAM-S score, the logarithm of 101 minus the percentage peripheral oxygen saturation, systolic blood pressure, and the log2 transformed C-reactive protein. This analysis was done on the 418 participants for which it was known whether the white blood cell count was abnormal or not. Missing values were imputed using a Bernoulli trial based on the probability of abnormal white blood cell count as estimated by the logistic regression model above.

Then, we imputed PCT (77 missing values). 119/348 participants had the lowest PCT value of 0.025. Therefore, we started by imputing whether PCT was higher than 0.025. This was done with a similar procedure as above, but with abnormal white blood cell count as an additional covariate (after it had been imputed). This analysis was done on the 348 participants for which the PCT value was not missing. The result was that 39 of the 77 missing PCT values were imputed as 0.025, and 38 as >0.025. For these 38 participants, we imputed the PCT value using a linear regression analysis with the same covariates and with log(log(PCT+1) as outcome. This analysis was done on the 229 participants with a real PCT value above 0.025. The missing values were imputed by the expected value based on the linear regression model, after back transforming to the original PCT scale (i.e. exp(exp(expected value))-1).
