# Supplementary material for: Recognition of Serious Infections in the Elderly Visiting the Emergency Department: The Development of a Diagnostic Prediction Model (ROSIE)
Source: Geriatrics (Basel). 2025 Apr 25;10(3):60. doi: 10.3390/geriatrics10030060 (PMC12101360; doi:10.3390/geriatrics10030060)
Supplement: Supplementary file 1 [file geriatrics-10-00060-s001.zip › Appendix F Risk calculation using the ROSIE model.pdf]

Appendix F: Risk calculation using the ROSIE model, elaborated example

Consider a patient with a systolic blood pressure of 100mmHg, an oxygen saturation of 92% and a CRP level of 77mg/L.

1. The regression formula (called the linear predictor) is calculated as -2.3898 + (100 * -0.0105) + (Log(101-92) * 0.9652) + (log2(77) * 0.2792) + (spline_term * 0.3782).

2. We have to calculate the spline term. This equals (max(0,(log2(77)-2.315868)3 - max(0,(log2(77)-6.195741)3 * (7.939331 – 2.315868) / (7.939331 – 6.195741) + max(0,(log2(77)-7.939331)3 * (6.195741 – 2.315868) / (7.939331 – 6.195741)) / (7.939331 – 2.315868)2 = 1.950196615.

3. Using the value for the spline term, the linear predictor equals 1.1683 (rounded at 4 decimals).

4. This is transformed into a risk estimate using 1 / (1 + exp(-1 * linear_predictor)), yielding an estimated risk of 0.76 or 76%.
