# Supplementary material for: Recognition of Serious Infections in the Elderly Visiting the Emergency Department: The Development of a Diagnostic Prediction Model (ROSIE)
Source: Geriatrics (Basel). 2025 Apr 25;10(3):60. doi: 10.3390/geriatrics10030060 (PMC12101360; doi:10.3390/geriatrics10030060)
Supplement: Supplementary file 1 [file geriatrics-10-00060-s001.zip › Appendix G R code for the ROSIE prediction model.pdf]

Appendix G: R-code for the ROSIE prediction model

```
##############################
##############################
## PREDICTION MODEL ROSIE   ##
##############################
##############################
# Thomas Struyf, Ben Van Calster, KU Leuven
# April 2024

###############################
# LOAD PACKAGES AND FUNCTIONS #
###############################
library(DescTools)
library(gmodels)
library(plyr)
library(rms) # logistic regression, restricted cubic splines
library(logistf) # LR using Firth's correction
library(pmsampsize)
source("C:\\Ben\\Statistics\\auc.nonpara.mw.R")
#library(auRoc) # AUC/c statistic using 95% CI based on the logit
transform
library(CalibrationCurves) # Calibration
library(boot) # bootstrapping, logit
library(locfit) # expit
library(ggplot2)
library(rmda) # Net Benefit and Decision Curve Analysis

fastAUC <- function(p, y) {
  x1 = p[y==1]; n1 = length(x1);
  x2 = p[y==0]; n2 = length(x2);
  r = rank(c(x1,x2))
  auc = (sum(r[1:n1]) - n1*(n1+1)/2) / n1 / n2
```

```
  return(auc)
}


###############
# DATA IMPORT #
###############

library(haven)
rosie <- read_sas("C:/Ben/ROSIE studie/masterfile_rosie_ed.sas7bdat", NULL)
#View(rosie)

rosie = rosie[,c("Y","O2_Saturation","CRP","PCT","HeartFreq","BreathFreq", "Systolic_BP", "Body_temperature","Abnormal_WBC", "CAM_S_score", "Age")]

####################
# DATA PREPARATION #
####################

# Make extra variable to model continuous variables using RCS with 3 knots (only needed on CC dataset)
# Log transform positively skewed CRP (it works better like that for spline modeling)

rosie$lO2_Saturation = log(101-rosie$O2_Saturation)
rosie$l2CRP = log2(rosie$CRP+1)
rosie$l2PCT = log2(rosie$PCT+1)
rosie$l2HeartFreq = log2(rosie$HeartFreq)
rosie$l2BreathFreq = log2(rosie$BreathFreq)

rosie$systolicrcs = rcspline.eval(rosie$Systolic_BP,nk=3)
rosie$lo2saturationrcs = rcspline.eval(rosie$lO2_Saturation,nk=3)
```

```
rosie$l2CRPrcs = rcspline.eval(rosie$l2CRP,nk=3)

rosie$Agercs  = rcspline.eval(rosie$Age,nk=3)
rosie$Bodyrcs  = rcspline.eval(rosie$Body_temperature,nk=3)
rosie$l2Heartrcs  = rcspline.eval(rosie$l2HeartFreq,nk=3)
rosie$l2Breathrcs  = rcspline.eval(rosie$l2BreathFreq,nk=3)

rosie$PCTb = 1*(rosie$PCT>0.025)
rosie$l2PCTrcs = rcspline.eval(rosie$l2PCT,nk=3)



##############
# IMPUTATION #
##############

# Abnormal white blood cell count

regimpAWBC = lrm(Abnormal_WBC ~ Y + Age + Body_temperature +
l2HeartFreq + l2BreathFreq + CAM_S_score + lO2_Saturation +
Systolic_BP + l2CRP,
                 data=rosie)
rosie$AWBCi = rbinom(dim(rosie)[1],1,predict(regimpAWBC,
newdata=rosie, type="fitted"))
rosie$Abnormal_WBCi =
ifelse(is.na(rosie$Abnormal_WBC),rosie$AWBCi,rosie$Abnormal_WBC)

# PCT: first impute whether PCT>0.025 or not, then impute PCT values
if >0.025

regimpPCTb = lrm(PCTb ~ Y + Age + Body_temperature + l2HeartFreq +
l2BreathFreq + CAM_S_score + lO2_Saturation +
                   Systolic_BP + l2CRP + Abnormal_WBCi, data=rosie)

rosie$PCTbi = rbinom(dim(rosie)[1],1,predict(regimpPCTb,
newdata=rosie, type="fitted"))
```

```
rosie$PCTbi = ifelse(is.na(rosie$PCTb),rosie$PCTbi,rosie$PCTb)

rosie$PCTi = rosie$PCT

rosie$PCTi[rosie$PCTbi==0]=0.025


rosie$llPCT = log(log(rosie$PCT + 1))

regimpPCT = lm(llPCT ~ Y + Age + Body_temperature + l2HeartFreq +
l2BreathFreq + CAM_S_score + lO2_Saturation +

                   Systolic_BP + l2CRP + Abnormal_WBCi,
data=rosie[rosie$PCTbi==1,])


rosie$PCTi[rosie$PCTbi==1 & is.na(rosie$PCT)] =
exp(exp(predict(regimpPCT, newdata=rosie[rosie$PCTbi==1 &
is.na(rosie$PCT),], type="response")))-1

rosie$l2PCTi = log2(rosie$PCTi+1)

rosie$l2PCTircs = rcspline.eval(rosie$l2PCTi,nk=3)



#########################################

# DESCRIPTIVE STATISTICS FOR PREDICTORS #

#########################################


Desc(rosie$O2_Saturation)

Desc(rosie$lO2_Saturation)

Desc(rosie$Systolic_BP)

Desc(rosie$CRP)

Desc(rosie$l2CRP)


Desc(rosie$PCT)

Desc(rosie$l2PCT)


Desc(rosie$Age)

Desc(rosie$Body_temperature)

Desc(log2(rosie$HeartFreq))

Desc(log2(rosie$BreathFreq))
```

```
Desc(rosie$CAM_S_score)

Desc(rosie$Abnormal_WBC)



###################################

# CORRELATIONS BETWEEN PREDICTORS #

###################################


# Spearman: used for two continuous/ordinal variables


round(cor(rosie[,c("Systolic_BP","O2_Saturation","CRP","PCT")],

          use="pairwise.complete.obs",method='spearman'),digits=2)



########################################

# UNIVARIABLE AUC'S FOR THE PREDICTORS #

########################################


# Manually ordered outcome labels to get AUC>=0.5


round(auc.nonpara.mw(rosie$Age[rosie$Y==0],rosie$Age[rosie$Y==1],method="pepe"), digits=2)

round(auc.nonpara.mw(rosie$Body_temperature[rosie$Y==1],rosie$Body_temperature[rosie$Y==0],method="pepe"), digits=2)

round(auc.nonpara.mw(rosie$HeartFreq[rosie$Y==1],rosie$HeartFreq[rosie$Y==0],method="pepe"), digits=2)

round(auc.nonpara.mw(rosie$BreathFreq[rosie$Y==1],rosie$BreathFreq[rosie$Y==0],method="pepe"), digits=2)

round(auc.nonpara.mw(rosie$Systolic_BP[rosie$Y==0],rosie$Systolic_BP[rosie$Y==1],method="pepe"), digits=2)

round(auc.nonpara.mw(rosie$O2_Saturation[rosie$Y==0],rosie$O2_Saturation[rosie$Y==1],method="pepe"), digits=2)

round(auc.nonpara.mw(rosie$CAM_S_score[rosie$Y==1],rosie$CAM_S_score[rosie$Y==0],method="pepe"), digits=2)

round(auc.nonpara.mw(rosie$CRP[rosie$Y==1],rosie$CRP[rosie$Y==0],method="pepe"), digits=2)
```

```
round(auc.nonpara.mw(rosie$PCT[rosie$Y==1 &
!is.na(rosie$PCT)],rosie$PCT[rosie$Y==0 &
!is.na(rosie$PCT)],method="pepe"), digits=2)

round(auc.nonpara.mw(rosie$Abnormal_WBC[rosie$Y==1 &
!is.na(rosie$Abnormal_WBC)],
                     rosie$Abnormal_WBC[rosie$Y==0 &
!is.na(rosie$Abnormal_WBC)],method="pepe"), digits=2)



####################################################################
##############
# UNIVARIABLE DESCRIPTIVE ASSESSMENT OF FUNCTIONAL FORM FOR
CONTINUOUS VARIABLES #
####################################################################
##############


# Systolic BP


systolicl = lrm(Y ~ Systolic_BP, data=rosie)
systolicrcs = lrm(Y ~ rcs(Systolic_BP,3), data=rosie)
systoliclp0 = predict(systolicl, newdata=c(1:229), se.fit=T)
systolicrcsp0 = predict(systolicrcs, newdata=c(1:229), se.fit=T)
systolicp =
as.data.frame(cbind(c(1:229),expit(cbind(systolicrcsp0[[1]],systolic
rcsp0[[1]]-
1.96*systolicrcsp0[[2]],systolicrcsp0[[1]]+1.96*systolicrcsp0[[2]]))
,
                            expit(systoliclp0[[1]])))


ggplot(systolicp, aes(x=V1)) +
  geom_line(aes(y = V2), color = "black", lwd=2) +
  geom_line(aes(y = V3), color="steelblue", linetype="twodash",
lwd=1.5) +
  geom_line(aes(y = V4), color="steelblue", linetype="twodash",
lwd=1.5) +
  geom_line(aes(y = V5), color = "black", linetype="dotted", lwd=1)
+
  labs(x = "Systolic blood pressure (mmHg)", y="Probability Y")+
```

```
  theme(axis.text=element_text(size=12),
axis.title=element_text(size=14,face="bold"))


# O2 saturation


o2saturationl = lrm(Y ~ O2_Saturation, data=rosie)

o2saturationl2 = lrm(Y ~ lO2_Saturation, data=rosie)

o2saturationrcs = lrm(Y ~ rcs(lO2_Saturation,3), data=rosie)

o2saturationlp0 = predict(o2saturationl, newdata=c(77:100),
se.fit=T)

o2saturationl2p0 = predict(o2saturationl2, newdata=log(101-
c(77:100)), se.fit=T)

o2saturationrcsp0 = predict(o2saturationrcs, newdata=log(101-
c(77:100)), se.fit=T)

o2saturationp =
as.data.frame(cbind(c(77:100),expit(cbind(o2saturationrcsp0[[1]],o2s
aturationrcsp0[[1]]-1.96*o2saturationrcsp0[[2]],


o2saturationrcsp0[[1]]+1.96*o2saturationrcsp0[[2]])),expit(o2saturat
ionlp0[[1]]),
                                    expit(o2saturationl2p0[[1]]))))


ggplot(o2saturationp, aes(x=V1)) +

  geom_line(aes(y = V2), color = "black", lwd=2) +

  geom_line(aes(y = V3), color="steelblue", linetype="twodash",
lwd=1.5) +

  geom_line(aes(y = V4), color="steelblue", linetype="twodash",
lwd=1.5) +

  geom_line(aes(y = V5), color = "black", linetype="dotted", lwd=1)
+

  geom_line(aes(y = V6), color = "black", linetype="dashed", lwd=1)
+

  labs(x = "O2 saturation", y="Probability Y")+

  theme(axis.text=element_text(size=12),
axis.title=element_text(size=14,face="bold"))


# CRP
```

```
CRPl = lrm(Y ~ CRP, data=rosie)

CRPl2 = lrm(Y ~ l2CRP, data=rosie)

CRPrcs = lrm(Y ~ rcs(l2CRP,3), data=rosie)

CRPlp0 = predict(CRPl, newdata=c(1:630), se.fit=T)

CRPl2p0 = predict(CRPl2, newdata=log2(c(1:630)), se.fit=T)

CRPrcsp0 = predict(CRPrcs, newdata=log2(c(1:630)), se.fit=T)

CRPp =
as.data.frame(cbind(c(1:630),expit(cbind(CRPrcsp0[[1]],CRPrcsp0[[1]]
-1.96*CRPrcsp0[[2]],

CRPrcsp0[[1]]+1.96*CRPrcsp0[[2]])),expit(CRPlp0[[1]]),expit(CRPl2p0[
[1]])))


ggplot(CRPp, aes(x=V1)) +

  geom_line(aes(y = V2), color = "black", lwd=2) +

  geom_line(aes(y = V3), color="steelblue", linetype="twodash",
lwd=1.5) +

  geom_line(aes(y = V4), color="steelblue", linetype="twodash",
lwd=1.5) +

  geom_line(aes(y = V5), color = "black", linetype="dotted", lwd=1)
+

  geom_line(aes(y = V6), color = "black", linetype="dashed", lwd=1)
+

  labs(x = "CRP", y="Probability Y")+

  theme(axis.text=element_text(size=12),
axis.title=element_text(size=14,face="bold"))



##########################

# MULTIVARIABLE MODELING #

##########################


# Backward selection (alpha 0.1) on spline terms
```

```
mod1 = lrm(Y ~ Systolic_BP + lO2_Saturation + l2CRP + systolicrcs +
lo2saturationrcs + l2CRPrcs, data=rosie)

mod2 = fastbw(mod1, rule="p", sls = 0.1, type="individual",
force=c(1:3))

mod3 = logistf(Y ~ ., flic=TRUE,
data=rosie[,c("Y",mod2$names.kept)])


# Odds ratios


exp(cbind(mod3$coefficients,mod3$coefficients-
1.96*sqrt(diag(mod3$var)),mod3$coefficients+1.96*sqrt(diag(mod3$var)
)))


# GET ESTIMATED RISKS FOR FINAL MODEL


estrisks = predict(mod3, type="response")

estrisks = as.data.frame(cbind(estrisks,rosie$Y))


# APPARENT C STATISTIC, CALIBRATION PLOT, NET BENEFIT, Sensitivity
and Specificity


appc1 =
auc.nonpara.mw(estrisks$estrisks[rosie$Y==1],estrisks$estrisks[rosie
$Y==0],method="pepe")


val.prob.ci.2(estrisks$estrisks,y=rosie$Y,smooth="loess",dostats =
T) # use loess fit, another option for flexible (nonlinear) modeling

appslope1 = glm(Y ~ logit(estrisks$estrisks),
family=binomial(link='logit'), data=rosie)$coefficients[2]


appdca1 <- decision_curve(V2~estrisks,
                              data = estrisks,
                              fitted.risk = TRUE,
                              thresholds = seq(0, 1, by = .05),
                              confidence.intervals = NA)

plot_decision_curve( list(appdca),
```

```
                     curve.names = c("ROSIE model"),
                     col = c("blue"),
                     ylim = c(-.005, 0.625), #set ylim
                     xlim = c(0,1),
                     lty = c(1), confidence.intervals = F,
                     standardize = FALSE, #plot Net benefit instead
of standardized net benefit
                     legend.position = "topright",xlab="Risk
threshold",cost.benefit.xlab="Harm to benefit ratio")
appnb1 = appdca1[[1]][1:21,c(1,6,7)]


appsesp1 =
round(matrix(data=c(binconf(sum(estrisks$estrisks[rosie$Y==1]>=0.2),
sum(rosie$Y==1),method="wilson"),

binconf(sum(estrisks$estrisks[rosie$Y==1]>=0.25),sum(rosie$Y==1),met
hod="wilson"),

binconf(sum(estrisks$estrisks[rosie$Y==1]>=0.3),sum(rosie$Y==1),meth
od="wilson"),

binconf(sum(estrisks$estrisks[rosie$Y==1]>=0.35),sum(rosie$Y==1),met
hod="wilson"),

binconf(sum(estrisks$estrisks[rosie$Y==1]>=0.4),sum(rosie$Y==1),meth
od="wilson"),

binconf(sum(estrisks$estrisks[rosie$Y==1]>=0.45),sum(rosie$Y==1),met
hod="wilson"),

binconf(sum(estrisks$estrisks[rosie$Y==1]>=0.5),sum(rosie$Y==1),meth
od="wilson"),

binconf(sum(estrisks$estrisks[rosie$Y==0]<0.2),sum(rosie$Y==0),metho
d="wilson"),

binconf(sum(estrisks$estrisks[rosie$Y==0]<0.25),sum(rosie$Y==0),meth
od="wilson"),

binconf(sum(estrisks$estrisks[rosie$Y==0]<0.3),sum(rosie$Y==0),metho
d="wilson"),
```

```
binconf(sum(estrisks$estrisks[rosie$Y==0]<0.35),sum(rosie$Y==0),meth
od="wilson"),

binconf(sum(estrisks$estrisks[rosie$Y==0]<0.4),sum(rosie$Y==0),metho
d="wilson"),

binconf(sum(estrisks$estrisks[rosie$Y==0]<0.45),sum(rosie$Y==0),meth
od="wilson"),

binconf(sum(estrisks$estrisks[rosie$Y==0]<0.5),sum(rosie$Y==0),metho
d="wilson")),
                    nrow=14,ncol=3,byrow=T),digits=2) # Apparent
sensitivity and specificity


# VIOLIN PLOTS OF ESTIMATED RISKS BY OUTCOME


estrisks$Yc[estrisks$V2==0] = "No serious infection"

estrisks$Yc[estrisks$V2==1] = "Serious infection"

p <- ggplot(estrisks, aes(x=Yc, y=estrisks)) +

  geom_violin() + theme(legend.position="none") + ylim(0,1) +

  labs(x = "Serious infection status", y="Estimated risk of serious
infection") +

  theme(axis.text=element_text(size=12),
axis.title=element_text(size=14,face="bold"))

p



# BOOTSTRAPPING FOR OPTIMISM CORRECTION, SELECTION FREQUENCY, AND
RISK INSTABILITY


nboot=200

simres = matrix(data = NA, nrow = nboot, ncol = 25)

rosie_boot = rosie[,c("Y", "Systolic_BP", "lO2_Saturation", "l2CRP",
"Age", "Body_temperature",

                      "l2HeartFreq", "l2BreathFreq", "CAM_S_score",
"Abnormal_WBCi",
```

```
                    "l2CRPrcs", "lo2saturationrcs", "systolicrcs",
"Agercs", "Bodyrcs", "l2Heartrcs", "l2Breathrcs")]

riskboot = as.data.frame(matrix(data = NA, nrow = 0, ncol = 425))


set.seed(1234)

for (bootnr in 1:nboot){


print(bootnr)


# Get bootstrap sample

train_data <-
rosie_boot[sample(row.names(rosie_boot),replace=TRUE),]


# Mimic the interim analysis after 342 patients (excluding PCT)

uniaucs =
c(max(fastAUC(p=train_data$Systolic_BP[1:342],y=train_data$Y[1:342])
,1-
(fastAUC(p=train_data$Systolic_BP[1:342],y=train_data$Y[1:342]))),


max(fastAUC(p=train_data$lO2_Saturation[1:342],y=train_data$Y[1:342]
),1-
(fastAUC(p=train_data$lO2_Saturation[1:342],y=train_data$Y[1:342])))
,

  max(fastAUC(p=train_data$l2CRP[1:342],y=train_data$Y[1:342]),1-
(fastAUC(p=train_data$l2CRP[1:342],y=train_data$Y[1:342]))),

  max(fastAUC(p=train_data$Age[1:342],y=train_data$Y[1:342]),1-
(fastAUC(p=train_data$Age[1:342],y=train_data$Y[1:342]))),


max(fastAUC(p=train_data$Body_temperature[1:342],y=train_data$Y[1:34
2]),1-
(fastAUC(p=train_data$Body_temperature[1:342],y=train_data$Y[1:342])
)),


max(fastAUC(p=train_data$l2HeartFreq[1:342],y=train_data$Y[1:342]),1
-(fastAUC(p=train_data$l2HeartFreq[1:342],y=train_data$Y[1:342]))),


max(fastAUC(p=train_data$l2BreathFreq[1:342],y=train_data$Y[1:342]),
1-
(fastAUC(p=train_data$l2BreathFreq[1:342],y=train_data$Y[1:342]))),
```

```
max(fastAUC(p=train_data$CAM_S_score[1:342],y=train_data$Y[1:342]),1
-(fastAUC(p=train_data$CAM_S_score[1:342],y=train_data$Y[1:342]))),

max(fastAUC(p=train_data$Abnormal_WBCi[1:342],y=train_data$Y[1:342])
,1-
(fastAUC(p=train_data$Abnormal_WBCi[1:342],y=train_data$Y[1:342]))))


# Select predictors with univariable AUC>=0.6

selvar =
c("Systolic_BP","lO2_Saturation","l2CRP","Age","Body_temperature","l
2HeartFreq","l2BreathFreq","CAM_S_score","Abnormal_WBCi")[uniaucs>=0
.6]


# Select rcs terms to consider based on univariable AUCs

selvarrcs = c()

selvarrcs = if (uniaucs[1]>=0.6) {append(selvarrcs,"systolicrcs")}
else if (uniaucs[1]<0.6) {selvarrcs}

selvarrcs = if (uniaucs[2]>=0.6)
{append(selvarrcs,"lo2saturationrcs")} else if (uniaucs[2]<0.6)
{selvarrcs}

selvarrcs = if (uniaucs[3]>=0.6) {append(selvarrcs,"l2CRPrcs")} else
if (uniaucs[3]<0.6) {selvarrcs}

selvarrcs = if (uniaucs[4]>=0.6) {append(selvarrcs,"Agercs")} else
if (uniaucs[4]<0.6) {selvarrcs}

selvarrcs = if (uniaucs[5]>=0.6) {append(selvarrcs,"Bodyrcs")} else
if (uniaucs[5]<0.6) {selvarrcs}

selvarrcs = if (uniaucs[6]>=0.6) {append(selvarrcs,"l2Heartrcs")}
else if (uniaucs[6]<0.6) {selvarrcs}

selvarrcs = if (uniaucs[7]>=0.6) {append(selvarrcs,"l2Breathrcs")}
else if (uniaucs[7]<0.6) {selvarrcs}


# do the modeling


mod5 = lrm(Y ~ ., data=train_data[,c("Y",selvar,selvarrcs)])

mod6 = fastbw(mod5, rule="p", sls = 0.1, type="individual",
force=c(1:length(selvar)))

mod7 = logistf(Y ~ ., flic=TRUE,
data=train_data[,c("Y",mod6$names.kept)])
```

```
# predict the values on the bootstrap data

predictboot3 <- predict(mod7,newdata=train_data)

predictboot3_risk <- predict(mod7,newdata=train_data,
type="response")


# predict the values on the original, unresampled data

predictorig3 <- predict(mod7, newdata = rosie_boot[,c(mod7$terms[-
1])])

predictorig3_risk <- predict(mod7, newdata =
rosie_boot[,c(mod7$terms[-1])],type="response")

riskboot = rbind.fill(riskboot,as.data.frame(t(predictorig3_risk)))


# return a vector of summary results

simres[bootnr,1:25] <- c(

    Cstat(predictboot3,train_data$Y), # AUC of bootstrap model on
bootstrap dataset

    Cstat(predictorig3,rosie_boot$Y), # AUC of bootstrap model on
original dataset

    glm(Y ~ predictboot3,  family=binomial(link='logit'),
data=train_data)$coefficients[2], # calibration slope bootstrap
dataset

    glm(Y ~ predictorig3,  family=binomial(link='logit'),
data=rosie_boot)$coefficients[2], # calibration slope original
dataset

    # Optimism in NB at relevant thresholds:

    ((sum(predictboot3_risk[train_data$Y==1]>=0.2) - (0.2/(1-
0.2))*sum(predictboot3_risk[train_data$Y==0]>=0.2)) -

       (sum(predictorig3_risk[rosie_boot$Y==1]>=0.2) - (0.2/(1-
0.2))*sum(predictorig3_risk[rosie_boot$Y==0]>=0.2)))/dim(rosie_boot)
[1],

    ((sum(predictboot3_risk[train_data$Y==1]>=0.25) - (0.25/(1-
0.25))*sum(predictboot3_risk[train_data$Y==0]>=0.25)) -

       (sum(predictorig3_risk[rosie_boot$Y==1]>=0.25) - (0.25/(1-
0.25))*sum(predictorig3_risk[rosie_boot$Y==0]>=0.25)))/dim(rosie_boo
t)[1],

    ((sum(predictboot3_risk[train_data$Y==1]>=0.3) - (0.3/(1-
0.3))*sum(predictboot3_risk[train_data$Y==0]>=0.3)) -
```

```
       (sum(predictorig3_risk[rosie_boot$Y==1]>=0.3) - (0.3/(1-
0.3))*sum(predictorig3_risk[rosie_boot$Y==0]>=0.3)))/dim(rosie_boot)
[1],

    ((sum(predictboot3_risk[train_data$Y==1]>=0.35) - (0.35/(1-
0.35))*sum(predictboot3_risk[train_data$Y==0]>=0.35)) -

       (sum(predictorig3_risk[rosie_boot$Y==1]>=0.35) - (0.35/(1-
0.35))*sum(predictorig3_risk[rosie_boot$Y==0]>=0.35)))/dim(rosie_boo
t)[1],

    ((sum(predictboot3_risk[train_data$Y==1]>=0.4) - (0.4/(1-
0.4))*sum(predictboot3_risk[train_data$Y==0]>=0.4)) -

       (sum(predictorig3_risk[rosie_boot$Y==1]>=0.4) - (0.4/(1-
0.4))*sum(predictorig3_risk[rosie_boot$Y==0]>=0.4)))/dim(rosie_boot)
[1],

    ((sum(predictboot3_risk[train_data$Y==1]>=0.45) - (0.45/(1-
0.45))*sum(predictboot3_risk[train_data$Y==0]>=0.45)) -

       (sum(predictorig3_risk[rosie_boot$Y==1]>=0.45) - (0.45/(1-
0.45))*sum(predictorig3_risk[rosie_boot$Y==0]>=0.45)))/dim(rosie_boo
t)[1],

    ((sum(predictboot3_risk[train_data$Y==1]>=0.5) - (0.5/(1-
0.5))*sum(predictboot3_risk[train_data$Y==0]>=0.5)) -

       (sum(predictorig3_risk[rosie_boot$Y==1]>=0.5) - (0.5/(1-
0.5))*sum(predictorig3_risk[rosie_boot$Y==0]>=0.5)))/dim(rosie_boot)
[1],

    # Optimism in sensitivity at relevant thresholds:


sum(predictboot3_risk[train_data$Y==1]>=0.2)/sum(train_data$Y==1)-
sum(predictorig3_risk[rosie_boot$Y==1]>=0.2)/sum(rosie_boot$Y==1),


sum(predictboot3_risk[train_data$Y==1]>=0.25)/sum(train_data$Y==1)-
sum(predictorig3_risk[rosie_boot$Y==1]>=0.25)/sum(rosie_boot$Y==1),


sum(predictboot3_risk[train_data$Y==1]>=0.3)/sum(train_data$Y==1)-
sum(predictorig3_risk[rosie_boot$Y==1]>=0.3)/sum(rosie_boot$Y==1),


sum(predictboot3_risk[train_data$Y==1]>=0.35)/sum(train_data$Y==1)-
sum(predictorig3_risk[rosie_boot$Y==1]>=0.35)/sum(rosie_boot$Y==1),


sum(predictboot3_risk[train_data$Y==1]>=0.4)/sum(train_data$Y==1)-
sum(predictorig3_risk[rosie_boot$Y==1]>=0.4)/sum(rosie_boot$Y==1),


sum(predictboot3_risk[train_data$Y==1]>=0.45)/sum(train_data$Y==1)-
sum(predictorig3_risk[rosie_boot$Y==1]>=0.45)/sum(rosie_boot$Y==1),
```

```
sum(predictboot3_risk[train_data$Y==1]>=0.5)/sum(train_data$Y==1)-
sum(predictorig3_risk[rosie_boot$Y==1]>=0.5)/sum(rosie_boot$Y==1),

    # Optimism in specificity at relevant thresholds:

sum(predictboot3_risk[train_data$Y==0]<0.2)/sum(train_data$Y==0)-
sum(predictorig3_risk[rosie_boot$Y==0]<0.2)/sum(rosie_boot$Y==0),

sum(predictboot3_risk[train_data$Y==0]<0.25)/sum(train_data$Y==0)-
sum(predictorig3_risk[rosie_boot$Y==0]<0.25)/sum(rosie_boot$Y==0),

sum(predictboot3_risk[train_data$Y==0]<0.3)/sum(train_data$Y==0)-
sum(predictorig3_risk[rosie_boot$Y==0]<0.3)/sum(rosie_boot$Y==0),

sum(predictboot3_risk[train_data$Y==0]<0.35)/sum(train_data$Y==0)-
sum(predictorig3_risk[rosie_boot$Y==0]<0.35)/sum(rosie_boot$Y==0),

sum(predictboot3_risk[train_data$Y==0]<0.4)/sum(train_data$Y==0)-
sum(predictorig3_risk[rosie_boot$Y==0]<0.4)/sum(rosie_boot$Y==0),

sum(predictboot3_risk[train_data$Y==0]<0.45)/sum(train_data$Y==0)-
sum(predictorig3_risk[rosie_boot$Y==0]<0.45)/sum(rosie_boot$Y==0),

sum(predictboot3_risk[train_data$Y==0]<0.5)/sum(train_data$Y==0)-
sum(predictorig3_risk[rosie_boot$Y==0]<0.5)/sum(rosie_boot$Y==0)
  ) # Net Benefit for thresholds (0.2:0.50) by 0.05

}

bootresults = list(simres,t(riskboot))

save(bootresults, file = "C:/Ben/ROSIE studie/bootresults.RData")
load ("C:/Ben/ROSIE studie/bootresults.RData")

# OPTIMISM CORRECTED PERFORMANCE

# C statistic
optc = mean(bootresults[[1]][,1]) - mean(bootresults[[1]][,2])
```

```
ccorr = appc1 - optc #optimism corrected c-statistic


# Calibration slope

optslope = mean(bootresults[[1]][,3]) - mean(bootresults[[1]][,4])

slopecorr = appslope1 - optslope


# Net Benefit, Sensitivity, Specificity

NBopt = colMeans(bootresults[[1]][,5:11])

Sensopt = colMeans(bootresults[[1]][,12:18])

Specopt = colMeans(bootresults[[1]][,19:25])


NBcorr = appnb1[5:11,2] - NBopt

Senscorr = round(cbind(appsesp1[1:7,1] - Sensopt, appsesp1[1:7,2] - Sensopt, appsesp1[1:7,3] - Sensopt), digits = 2)

Speccorr = round(cbind(appsesp1[8:14,1] - Specopt, appsesp1[8:14,2] - Specopt, appsesp1[8:14,3] - Specopt), digits = 2)


# Decision curve

plot(100*appdca1[[1]][22:42,1], col="white", xlim=c(0,100),ylim=c(0,0.55),

     ylab="Net Benefit", xlab="Decision threshold (% risk of serious infection)")

polygon(c(20,50,50,20), c(0,0,0.56,0.56), col = "lightgray", border=NA)

lines(100*appdca1[[1]][22:42,1], appdca1[[1]][22:42,6], type="l",lty=2,col="black")

lines(c(20,25,30,35,40,45,50), NBcorr, lwd=2,col="red")

abline(h=0,col="black")

lines(100*appdca1[[1]][1:21,1], appdca1[[1]][1:21,6], lwd=2, col="blue")

legend("topright", inset=.02, legend=c("ROSIE (uncorrected)", "ROSIE (corrected)",

                                       "Treat all", "Treat none"),

       col=c("blue","red","gray","black"), lty=c(1,1,2,1), lwd=c(1,2,1,1), cex=0.8)
```

```
# RISK INSTABILITY PLOT

meanrisk = rowMeans(bootresults[[2]])

meanriskr = rank(meanrisk)

plot(meanriskr,bootresults[[2]][,1],type="p", pch=".",col="gray",
cex=0.1, xlab="Patient", ylab="Estimated risk of Y", ylim=c(0,1))

for (i in 2:200){

  points(meanriskr,bootresults[[2]][,i], pch=".",col="gray",
cex=0.1)

}

points(meanriskr, meanrisk, pch=".", col="red", cex=2)




# The exploratory model with PCT as an additional predictor

mod1p = lrm(Y ~ Systolic_BP + lO2_Saturation + l2CRP + l2PCTi +
systolicrcs + lo2saturationrcs + l2CRPrcs + l2PCTircs, data=rosie)

mod2p = fastbw(mod1p, rule="p", sls = 0.1, type="individual",
force=c(1:4))

mod3p = logistf(Y ~ ., flic=TRUE,
data=rosie[,c("Y",mod2p$names.kept)])

estrisksp = predict(mod3p, type="response")

estrisksp = as.data.frame(cbind(estrisksp,rosie$Y))

appc1p =
auc.nonpara.mw(estrisksp$estrisksp[rosie$Y==1],estrisksp$estrisksp[r
osie$Y==0],method="pepe")


# BOOTSTRAPPING FOR EXPLORATORY MODEL WITH PCT

nboot=200

simresp = matrix(data = NA, nrow = nboot, ncol = 2)

rosie_boot = rosie[,c("Y", "Systolic_BP", "lO2_Saturation", "l2CRP",
"Age", "Body_temperature",
```

```
                      "l2HeartFreq", "l2BreathFreq", "CAM_S_score",
"Abnormal_WBCi", "l2PCTi",
                      "l2CRPrcs", "lo2saturationrcs", "systolicrcs",
"Agercs", "Bodyrcs", "l2Heartrcs", "l2Breathrcs", "l2PCTircs")]


set.seed(1234)
for (bootnr in 1:nboot){


  print(bootnr)


  # Get bootstrap sample
  train_data <-
rosie_boot[sample(row.names(rosie_boot),replace=TRUE),]


  # Mimic the interim analysis after 342 patients (with PCT)
  uniaucs =
c(max(fastAUC(p=train_data$Systolic_BP[1:342],y=train_data$Y[1:342])
,1-
(fastAUC(p=train_data$Systolic_BP[1:342],y=train_data$Y[1:342]))),

max(fastAUC(p=train_data$lO2_Saturation[1:342],y=train_data$Y[1:342]
),1-
(fastAUC(p=train_data$lO2_Saturation[1:342],y=train_data$Y[1:342])))
,

max(fastAUC(p=train_data$l2CRP[1:342],y=train_data$Y[1:342]),1-
(fastAUC(p=train_data$l2CRP[1:342],y=train_data$Y[1:342]))),

max(fastAUC(p=train_data$Age[1:342],y=train_data$Y[1:342]),1-
(fastAUC(p=train_data$Age[1:342],y=train_data$Y[1:342]))),

max(fastAUC(p=train_data$Body_temperature[1:342],y=train_data$Y[1:34
2]),1-
(fastAUC(p=train_data$Body_temperature[1:342],y=train_data$Y[1:342])
)),

max(fastAUC(p=train_data$l2HeartFreq[1:342],y=train_data$Y[1:342]),1
-(fastAUC(p=train_data$l2HeartFreq[1:342],y=train_data$Y[1:342]))),

max(fastAUC(p=train_data$l2BreathFreq[1:342],y=train_data$Y[1:342]),
```

```
1-
(fastAUC(p=train_data$l2BreathFreq[1:342],y=train_data$Y[1:342]))),

max(fastAUC(p=train_data$CAM_S_score[1:342],y=train_data$Y[1:342]),1
-(fastAUC(p=train_data$CAM_S_score[1:342],y=train_data$Y[1:342]))),

max(fastAUC(p=train_data$Abnormal_WBCi[1:342],y=train_data$Y[1:342])
,1-
(fastAUC(p=train_data$Abnormal_WBCi[1:342],y=train_data$Y[1:342]))),

max(fastAUC(p=train_data$l2PCTi[1:342],y=train_data$Y[1:342]),1-
(fastAUC(p=train_data$l2PCTi[1:342],y=train_data$Y[1:342]))))


  # Select predictors with univariable AUC>=0.6

  selvar =
c("Systolic_BP","lO2_Saturation","l2CRP","Age","Body_temperature","l
2HeartFreq","l2BreathFreq","CAM_S_score","Abnormal_WBCi","l2PCTi")[u
niaucs>=0.6]


  # Select rcs terms to consider based on univariable AUCs

  selvarrcs = c()

  selvarrcs = if (uniaucs[1]>=0.6) {append(selvarrcs,"systolicrcs")}
else if (uniaucs[1]<0.6) {selvarrcs}

  selvarrcs = if (uniaucs[2]>=0.6)
{append(selvarrcs,"lo2saturationrcs")} else if (uniaucs[2]<0.6)
{selvarrcs}

  selvarrcs = if (uniaucs[3]>=0.6) {append(selvarrcs,"l2CRPrcs")}
else if (uniaucs[3]<0.6) {selvarrcs}

  selvarrcs = if (uniaucs[4]>=0.6) {append(selvarrcs,"Agercs")} else
if (uniaucs[4]<0.6) {selvarrcs}

  selvarrcs = if (uniaucs[5]>=0.6) {append(selvarrcs,"Bodyrcs")}
else if (uniaucs[5]<0.6) {selvarrcs}

  selvarrcs = if (uniaucs[6]>=0.6) {append(selvarrcs,"l2Heartrcs")}
else if (uniaucs[6]<0.6) {selvarrcs}

  selvarrcs = if (uniaucs[7]>=0.6) {append(selvarrcs,"l2Breathrcs")}
else if (uniaucs[7]<0.6) {selvarrcs}

  selvarrcs = if (uniaucs[10]>=0.6) {append(selvarrcs,"l2PCTircs")}
else if (uniaucs[7]<0.6) {selvarrcs}
```

```
  # do the modeling (THE CODE DID NOT CONSIDER VARIABLE SELECTION OF
RCS TERMS!)

  # BVC modeling code


  mod5 = lrm(Y ~ ., data=train_data[,c("Y",selvar,selvarrcs)])

  mod6 = fastbw(mod5, rule="p", sls = 0.1, type="individual",
force=c(1:length(selvar)))

  mod7 = logistf(Y ~ ., flic=TRUE,
data=train_data[,c("Y",mod6$names.kept)])


  # predict the values on the bootstrap data

  predictboot3 <- predict(mod7,newdata=train_data)

  predictboot3_risk <- predict(mod7,newdata=train_data,
type="response")


  # predict the values on the original, unresampled data

  predictorig3 <- predict(mod7, newdata = rosie_boot[,c(mod7$terms[-
1])])

  predictorig3_risk <- predict(mod7, newdata =
rosie_boot[,c(mod7$terms[-1])],type="response")


  # return a vector of summary results

  simresp[bootnr,1:2] <- c(

    Cstat(predictboot3,train_data$Y), # AUC of bootstrap model on
bootstrap dataset

    Cstat(predictorig3,rosie_boot$Y)) # AUC of bootstrap model on
original dataset


}


save(simresp, file = "C:/Ben/ROSIE
studie/bootresults_withPCT.RData")

load ("C:/Ben/ROSIE studie/bootresults_withPCT.RData")



# OPTIMISM CORRECTED PERFORMANCE
```

```
# C statistic

optcp = mean(simresp[,1]) - mean(simresp[,2])

ccorrp = appc1p - optcp #optimism corrected c-statistic
```
